# Selective Autooxidation of Ethanol over Titania-Supported Molybdenum Oxide Catalysts: Structure and Reactivity

**DOI:** 10.1002/adsc.201000841

**Published:** 2012-04-19

**Authors:** Carlos Caro, K Thirunavukkarasu, M Anilkumar, N R Shiju, Gadi Rothenberg

**Affiliations:** a Van 't Hoff Institute for Molecular Sciences, University of AmsterdamScience Park 904, 1090 GD Amsterdam, The Netherlands, Fax: (+31)-(0)20-525-5604; e-mail: n.r.shiju@uva.nl or g.rothenberg@uva.nl; b Department of Physical, Chemical and Natural Systems, Universidad Pablo de Olavide, Carretera de Utrera Km 141013 Sevilla, Spain; c National Centre for Catalysis Research, Indian Institute of TechnologyMadras, Chennai – 600 036, India; d Department of Chemistry, National University of Singapore3-Science Drive 3, Singapore 117543

**Keywords:** acetaldehyde, heterogeneous catalysis, molybdenum oxide, sustainable chemistry, UV-visible spectroscopy

## Abstract

We study the selective catalytic oxidation of ethanol with air as a sustainable alternative route to acetaldehyde. The reaction is catalysed by molybdenum oxide supported on titania, in a flow reactor under ambient pressure. High selectivity to acetaldehyde (70%–89%, depending on the Mo loading) is obtained at 150 °C. Subsequently, we investigate the structure/performance relationship for various molybdenum oxide species using a combination of techniques including diffuse reflectance UV-visible, infrared, X-ray photoelectron spectroscopies, X-ray diffraction and temperature programmed reduction. As their surface density increases, the monomeric molybdenum oxide species undergo two-dimensional and three-dimensional oligomerisation. This results in polymolybdates and molybdenum oxide crystallites. Importantly, the ethanol oxidation rate depends not only on the overall molybdenum loading and dispersion, but also on the type of molybdenum oxide species prevalent at each surface density and on the domain size. As the molybdenum oxide oligomerisation increases, electron delocalisation becomes easier. This lowers the absorption edge energy and increases the reaction rate.

## Introduction

While much research effort in industry and academia is now directed towards sustainable chemicals production and biomass utilisation, petroleum-based processes still account for over 90% of carbon-containing chemicals.^1^ Most of these processes are highly efficient, reaping the benefits of many decades of optimisation and investment. This means that if we really plan on introducing alternative routes to bulk chemicals starting from renewable resources, these must be simple and straightforward. Otherwise, they will not compete with the current technology on an economic basis.

One good example is the production of acetaldehyde (ethanal, CH_3_CHO), a key bulk chemical for the production of acetic acid, pyridine and pyridine bases, peracetic acid, pentaerythritol, butylene glycol, and chloral, with a worldwide production of 10^6^ tons/year in 2003.^2^ Currently, it is produced chiefly by the Hoescht–Wacker process [Eq. (1)], *via* oxidation of ethylene in the liquid phase catalysed by palladium and copper.^3^,^4^ This is an efficient and robust process, albeit petroleum-based. In this case, however, there is an alternative and renewable feedstock, bioethanol, that is already available on an industrial scale (worldwide bioethanol production topped 40 MMT in 2005).^5^ Thus, the air oxidation (autooxidation) of ethanol to acetaldehyde [Eq. (2)] could be a viable alternative, provided that a suitable catalyst is found.1
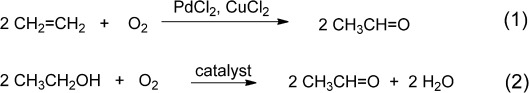


There are few studies on this catalytic system. Mixed oxides containing molybdenum and tin were used for vapour-phase ethanol oxidation. At higher oxygen pressures and temperatures of ∼250 °C, the main product was acetic acid.^6^ At 150 °C, acetaldehyde was the predominant product (70% selectivity).^7^ Cerium doping to Mo–Sn–O catalysts increased the activity and selectivity for ethanol partial oxidation,^8^ while mixed Nb–Mo–V oxides dispersed on titania gave a 95% selectivity to acetic acid, again at high pressure and temperature (16 bar, 237 °C).^9^ Vanadia catalysts on various supports were also applied in gas-phase and aqueous-phase ethanol oxidation.^10^,^11^ These catalysts favoured the formation of acetaldehyde below 200 °C. In contrast, V_2_O_5_ supported on TiO_2_ and immobilised on clay was highly selective to acetic acid (97%), even at relatively moderate temperatures and pressures (180 °C and 1.7 bar).^12^ In another recent work, acetic acid was selectively obtained by liquid-phase ethanol oxidation over Au/MgAl_2_O_4_ at 180 °C and 35 bar air.^13^ However, at ethanol concentrations above 60%, the same catalyst gave primarily ethyl acetate *via* acetaldehyde.^14^ In another work, the effect of the support was studied for ethanol oxidation.^15^ The large performance differences for very similar metal oxides in different structural environments show that the active species’ local structure strongly affects the oxidation mechanism.

Here we report the local structure-activity relationship of MoO_x_/TiO_2_ catalysts for vapour-phase autooxidation of ethanol. We use MoO_x_/TiO_2_, rather than mixed oxides, since this enables better control of the local structure around the Mo sites. After synthesising MoO_x_/TiO_2_ catalysts with different Mo surface densities by wet impregnation, we test them in ethanol oxidation under flow conditions. Using UV-visible diffuse reflectance spectroscopy (UV-Vis DRS), we examine the effect of surface density on the MoO_x_ domain structure, and outline the structural requirements for selective ethanol oxidation on MoO_x_/TiO_2_ catalysts.

## Results and Discussion

### Catalyst Preparation

A series of five MoO_x_/TiO_2_ catalysts **1**–**5**, with Mo loadings of 2%–14%, were prepared by incipient wetness impregnation of titania with aqueous solutions of ammonium molybdate (detailed procedures given in the Experimental Section). We opted for low Mo loadings, thus keeping the Mo dispersion on the support surface high. Table 1 shows the composition, surface characteristics and activity data of these catalysts as well as data for four reference catalysts.

**Table 1:** Nomenclature and properties of MoO_x_-TiO_2_ catalysts

Catalyst	Mo loading [wt%][a]	Mo surface density [atoms/nm^2^][b]	Absorption edge energy [AEE, eV][c]	Ethanol conversion [mol%][d]	Acetaldehyde selectivity [mol%][e]
**1**	1.96	1.11	3.61	10.1	88.5
**2**	3.91	2.23	3.47	19.8	85.5
**3**	6.96	3.97	3.19	29.8	78.2
**4**	10.0	5.71	3.06	36.1	79.1
**5**	13.9	7.94	2.89	41.4	69.8
MoO_3_/TiO_2_[f]	5.3 (MoO_3_)	4.8	n/d	17	94
VO_x_/TiO_2_/SiO_2_[g]	2.3 (V_2_O_5_)	0.9	n/d	∼15	∼95
V_2_O_5_/TiO_2_[h]	15 (V_2_O_5_)	7.7	n/d	∼85	∼95
Mo–Ce–Sn–O[i]	8% Mo, 1%Ce	4.03	n/d	∼81	∼75

[a] Nominal loading.

[b] Calculated from the Mo loading and BET surface area; provides a theoretical estimate of MoO_x_ species per unit area (nm^−2^) of the catalyst.

[c] Calculated from UV-Vis DRS spectra.

[d] Continuous steady-state conversion at 150 °C observed over 1 h; determined by GC as moles of ethanol reacted per mole of ethanol fed.

[e] GC selectivity determined as moles of a particular product formed per mole of all products formed.

[f] Ethanol:O_2_:H_2_O:He:N_2_=1:3.3:10:35.3:0.34; total pressure: 1.6 MPa, *T*=200 °C.^9^

[g] Molar composition: 1.4% EtOH vapour, 28.0% O_2_ and balance N_2_. W/F=11.7 g catalyst×h mol^−1^ of ethanol. *T*=140 °C; surface density based on V atoms/nm^2^; VO_x_ denotes that the exact nature of vanadium containing species is unclear.^16^

[h] *P*=2.7 bar, GHSV=25000 h^−1^, O_2_/ethanol mole ratio=3.3; *T*=175 °C; surface density based on V atoms per square nm.^11^

[i] 3% ethanol/air (vol.%) mixture, space velocity=11520 h^−1^; *T*=150 °C.^8^

### Catalytic Activity Studies

The ethanol oxidation activities of catalysts **1**–**5** were studied using a vapour-phase fixed-bed reactor. Earlier ethanol oxidation studies were conducted at high temperatures and/or pressures, leading to the predominant production of acetic acid. Conversely, we ran our reactions at a lower temperature, 150 °C, and using air instead of oxygen. In a typical reaction [Eq. (2)], the catalyst bed was first pre-treated at 400 °C and then cooled to 150 °C. The reaction was then started by feeding ethanol and air. Each reaction was run until a steady-state was reached (typically 2 h) and then continued for another hour. Control experiments run at 150 °C with a blank reactor ruled out the possibility of a homogeneous (gas/vapour phase) reaction.

**Figure 1:**
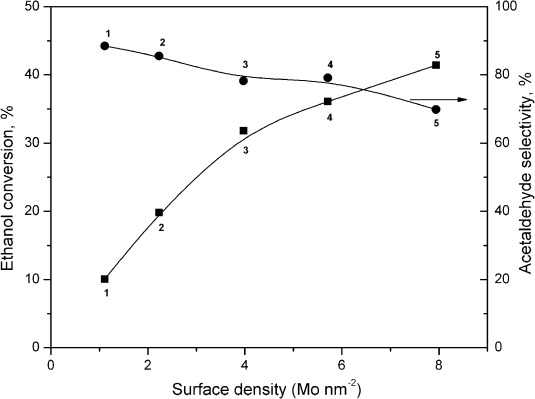
Catalytic performance of MoO_x_/TiO_2_ catalysts as a function of Mo surface density. Catalyst numbers (as in Table 1) are indicated in the figure. *Reaction conditions:* 150 °C, ethanol WHSV=10.5 h^−1^_,_ 50 mL min^−1^ air.

The major product observed on all MoO_*x*_/TiO_2_ catalysts at 150 °C was acetaldehyde (Table 1), with acetic acid as by-product, in agreement with the results of Li et al.^9^ Carbon oxides were not observed. Increasing the Mo surface density led to higher ethanol conversion (Figure 1). This shows that the effectiveness of MoO_x_/TiO_2_ catalysts depends on the nature of surface MoO_x_ species. In general, different types of species can form when a transition metal oxide is deposited on a support depending on the treatment temperature, loading, precursors and nature of support. Such species vary in their reactivity which ultimately determines the catalytic performance. Notably, a similar sensitivity to structural features was observed elsewhere also for the analogous V_2_O_5_ catalysts: Acetaldehyde was the major product when V_2_O_5_/MCM-41 was used,^10^ whereas the reaction was highly selective to acetic acid over V_2_O_5_/TiO_2_/clay.^12^

### Catalyst Characterisation

**Figure 2:**
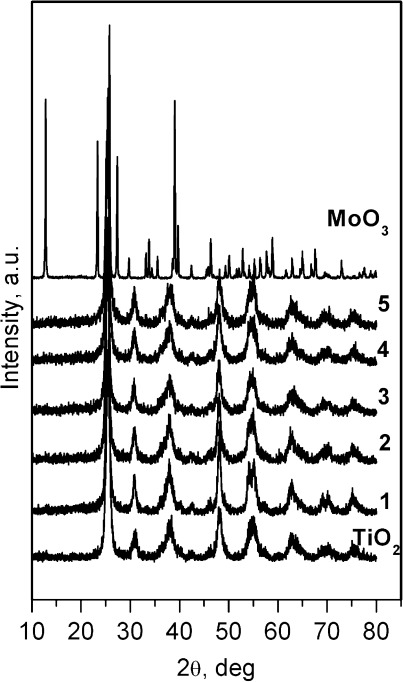
XRD patterns of the catalysts **1**–**5**, MoO_3_ and TiO_2_ samples.

We compared the XRD patterns of the catalysts **1–5** with those of pure MoO_3_ and the support TiO_2_ (Figure 2). Standard data were used to identify the species present in the samples. TiO_2_ support contains both anatase (JCPDS No. 21-1272) and brookite phases (JCPDS No. 29-1360). The XRD results show only peaks corresponding to titania for catalysts **1**–**5**, indicating a high dispersion of molybdenum oxide on the support. The dominant phase in TiO_2_ support and catalysts is anatase. From the broadness of the peaks we conclude that TiO_2_ as well as catalysts **1–5** have a high surface area, in agreement with the results shown in Table 1.

**Figure 3:**
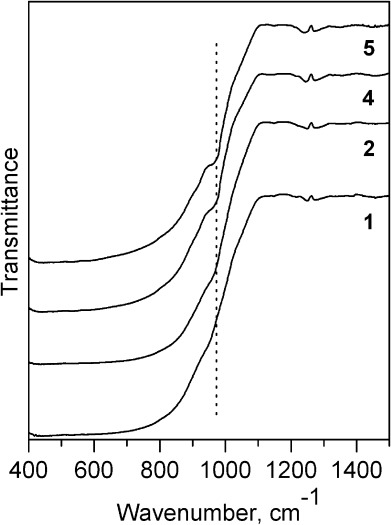
FT-IR spectra of the catalysts.

Generally, the IR band of Mo=O in crystalline MoO_3_ appears at 1000 cm^−1^ due to the stretching vibration mode. No bands are observed in the IR spectra of catalysts with low Mo loadings (Figure 3). At higher loadings, weak bands are observed close to the above value. We conclude that molybdenum oxide is highly dispersed at lower loadings.

**Figure 4:**
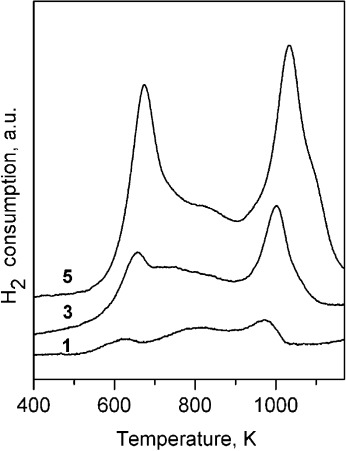
TPR profiles of catalysts **1**, **3** and **5**.

Figure 4 shows the TPR profiles of the catalysts **1**, **3**, and **5**. The peak area and H_2_ consumption increased with increasing Mo loading, as expected. It is reported that TPR of bulk MoO_3_ shows two major peaks.^17^ This may be attributed to the MoO_3_ reduction in two steps, such as MoO_3_→MoO_2_→Mo. The TPR profiles of catalysts **1**, **3** and **5** show that the reduction occurs in two stages. The first reduction step occurs at much lower temperature than the first reduction step of bulk MoO_3_. However, the difference in maximum temperatures (*T*_max_) is not that high for the second reduction. In general, when an oxide is supported on a high surface area support, *T*_max_ decreases considerably due to the interaction with the support. Our catalysts contain different Mo species, and these are reduced at different temperatures, based on the strength with which they are attached on the support. Note that the reduction of supported metal oxides is influenced by the coordination of the metal cation, state of aggregation and the crystal plane of the support on which the oxide is attached.^18^

**Figure 5:**
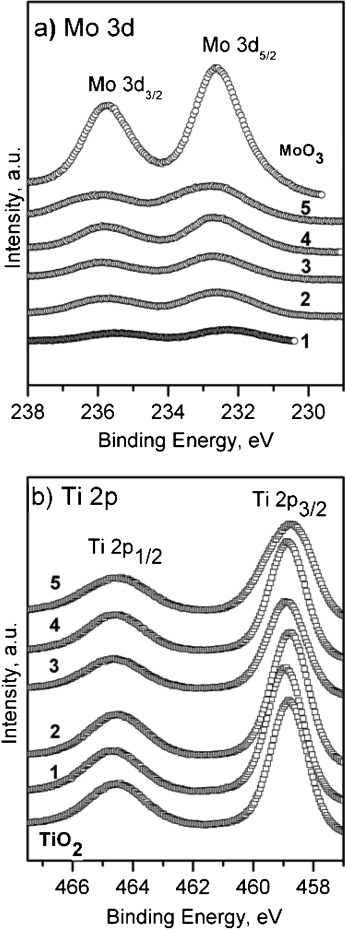
X-ray photoelectron spectra of the catalysts in the **a**) Mo 3*d* and **b**) Ti 2*p* regions.

Figure 5 shows the Mo 3*d* and Ti 2*p* XP spectra. Full width half maximum (FWHM) values for Mo 3*d*_5/2_ and Ti 2*p*_3/2_ peaks were calculated by deconvoluting the corresponding spectra. Table 2 shows these values together with the binding energies (BE) for Mo 3*d*_5/2_, Ti 2*p*_3/2_ and O 1*s*, Mo 3*d*/Ti 2*p* intensity ratio and the surface ratio of Mo and Ti. The Mo 3*d* spectrum exhibits a broad doublet corresponding to the 3*d*_5/2_ and 3*d*_3/2_ states (spin-orbit coupling of a 3*d* state). The binding energy of the Mo 3*d*_5/2_ peak of catalysts **1**–**5** is close to that for bulk MoO_3_, indicating the presence of mainly Mo(VI) on the catalysts. Sharp Mo 3*d* peaks were observed for MoO_3_, however, we obtained broadened peaks for catalysts **1**–**5**. In the literature, peak broadening has been attributed to the presence of more than one Mo(VI) species or to electron transfer reactions between the support and oxide or to charging.^19^–^22^ In general, Mo 3*d*_5/2_ and Ti 2*p*_3/2_ peaks showed broadening with increasing MoO_3_ loading. The higher BE values may reflect MoO_3_ crystallite formation on the TiO_2_ surface. Note that although a weak band attributable to MoO_3_ was observed for higher loadings in FTIR, no MoO_3_ peaks were detected in XRD. Hence, these 3-dimensional crystallites of MoO_3_ must be below the detection limit.

**Table 2:** XPS data of the catalysts

Catalyst	Mo 3*d*_5/2_		Ti 2*p*_3/2_ (eV)		O 1*s* (eV)		Mo3*d*/Ti2*p*	% Mo on surface
	BE[a] [eV]	FWHM[b] [eV]	BE [eV]	FWHM [eV]	BE [eV]	FWHM [eV]		
TiO_2_	–	–	458.791	1.4299	529.950	1.3640	–	–
**1**	232.209	1.9029	458.908	1.4244	529.953	1.3892	0.1605	11.60
**2**	232.531	1.9567	458.780	1.4481	530.053	1.4127	0.3096	20.21
**3**	232.673	1.9656	458.885	1.5025	530.029	1.4655	0.4026	24.69
**4**	232.631	1.8993	458.849	1.4191	530.055	1.4363	0.4874	28.42
**5**	232.775	2.2456	458.801	1.7581	529.952	1.5776	0.5879	32.26
MoO_3_	232.599	1.6143	–	–	530.201	1.4850	–	100

[a] BE = binding energy.

[b] FWHM = full width at half maximum.

**Figure 6:**
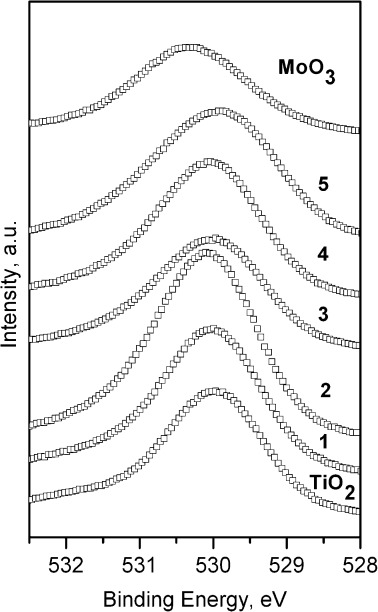
X-ray photoelectron spectra of the catalysts in the O 1*s* region.

Figure 6 shows the XP spectra of the O 1*s* region of the catalysts, MoO_3_ and TiO_2_. At lower loadings, the O 1*s* peak shape looks like that of TiO_2_. At higher loadings, the peaks resemble the shape of MoO_3_ O 1*s* peak. This further confirms the formation of 3-dimensional MoO_3_ species on the surface.

Diffuse reflectance UV-Vis spectra were measured for catalysts **1–5** after calcinations. All five catalysts showed main absorption features at energies ranging from 2.5 eV to 6 eV due to ligand-to-metal charge transfers from the oxygen *2p* orbital to the molybdenum *d* orbital.

The spectra were then rigorously analysed at the incipient absorption region using the optical absorption edge energy (AEE) values. This is the minimum energy required to excite an electron from the highest occupied molecular orbital (HOMO) of a lattice oxygen atom to the lowest unoccupied molecular orbital (LUMO) in a metal cation. The average domain size of nanostructured supported transition metal oxides is strongly related to their AEE, just like the size of semiconductor nanoparticles depends on their electronic excitation energies.^23^

**Figure 7:**
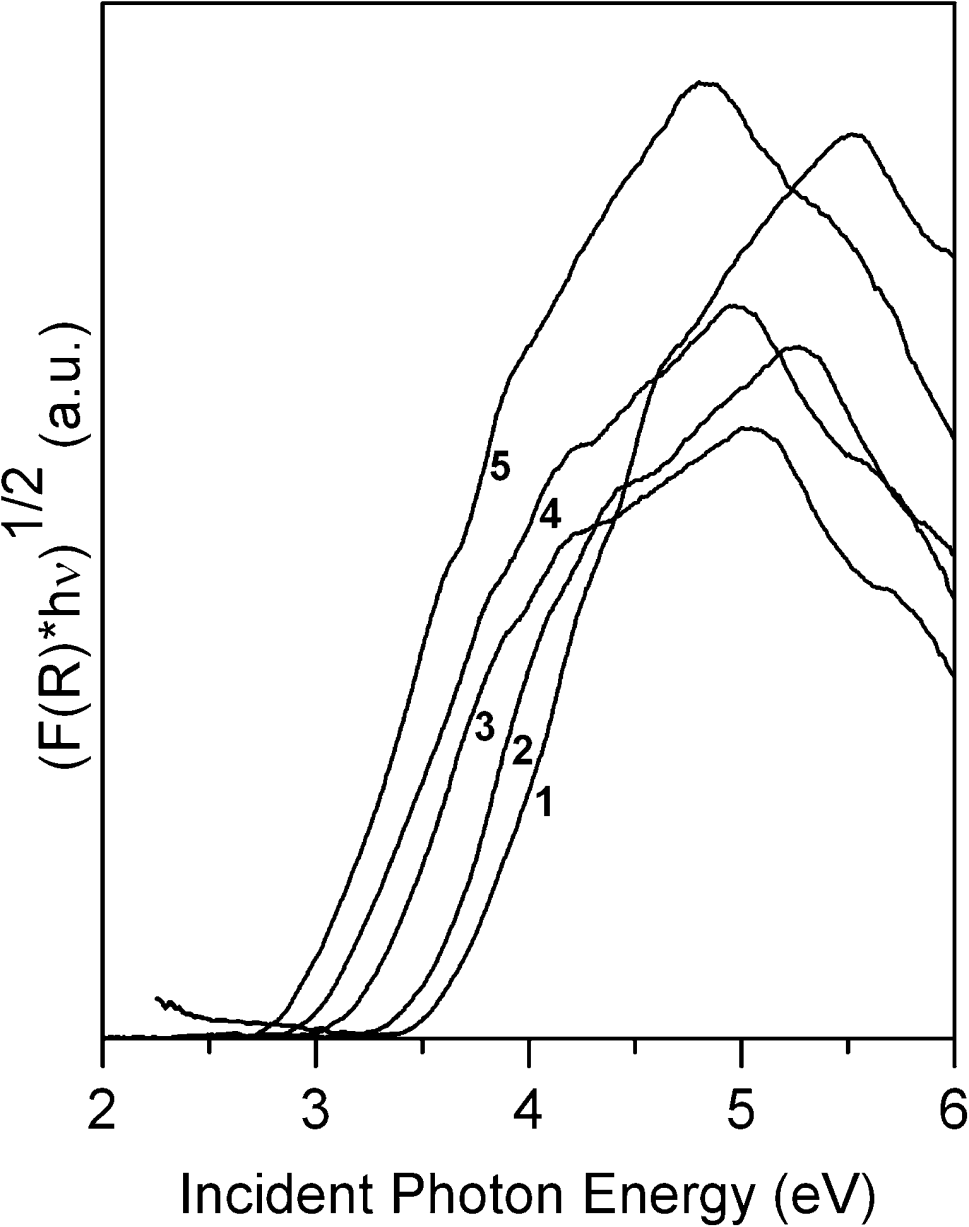
UV-Vis absorption spectra of catalysts **1**–**5**.

We calculated the AEE values of MoO_x_-TiO_2_ catalysts using the model typically applied for indirect-allowed HOMO–LUMO transitions.^24^–^29^ In this edge energy analysis, reflectance measurements were first converted into pseudo-absorbance units using the Kubelka–Munk transform. The absorption expressions, multiplied by incident photon energies raised to the appropriate power, were then plotted *vs.* the energy (Figure 7).^17^,^30^

**Figure 8:**
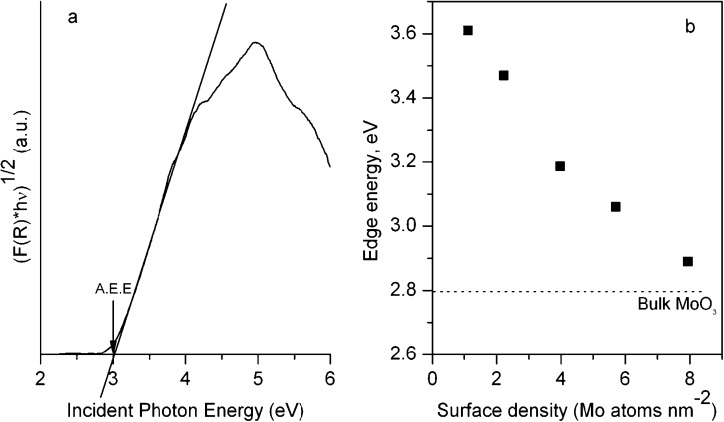
(**a**) Edge energy determination by linear extrapolation of absorption onset shown for catalyst **4**; (**b**) edge energy variation as a function of Mo surface density.

The AEE values were then determined by finding the *x*-intercept of the tangent close to the absorption onset in the [F(R_∞_)hν]^1/η^ versus hν plots, where F(R_∞_) is the Kubelka–Munk function, R_∞_ is the reflectance at infinite sample thickness, hν is the incident photon energy, and η=2 for indirect-allowed transitions. Figure 8a exemplifies this procedure for catalyst **4**.

**Figure 9:**
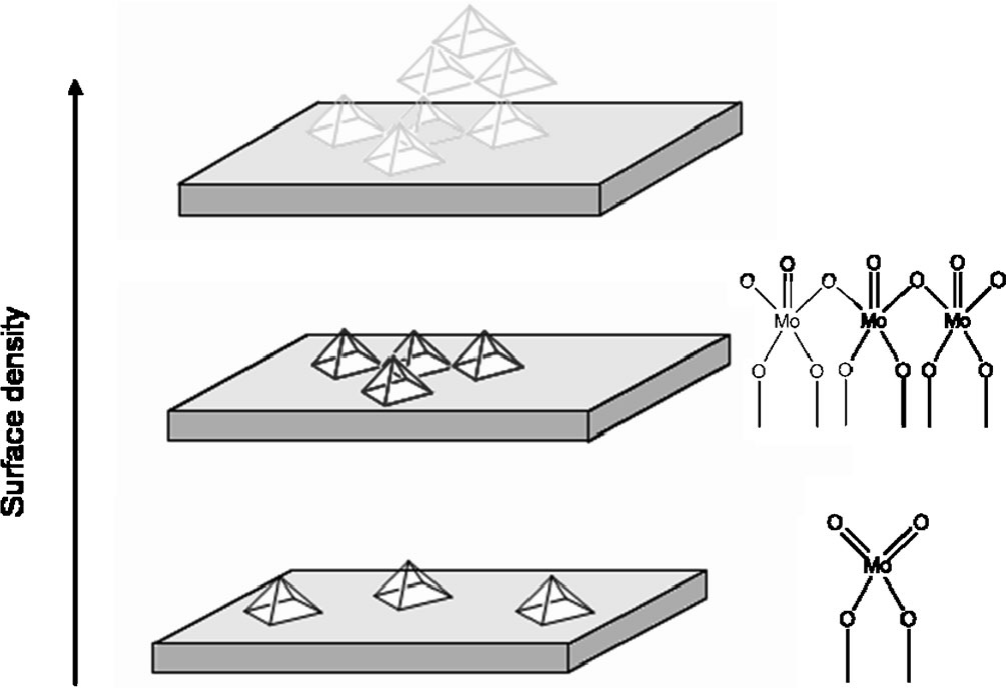
Cartoon showing the evolution of MoO_x_ species from monomeric to oligomeric structures with increasing surface density. Monomeric species (pyramids on the lower level) first grow two-dimensionally (pyramids on the middle level) and then three-dimensionally (pyramids on the upper level). The first two types of MoO_x_ species are depicted in the bottom part. The pyramidal shapes are shown for illustrative purposes only.

The calculated AEE values are plotted in Figure 8b, showing a decreasing trend with increasing surface density. At the lowest surface density (catalyst **1**; 1.1 Mo atoms/nm^2^), the AEE equals 3.6 eV. This value is lower than that reported for isolated Mo species (*cf.* 4.3 eV for Na_2_MoO_4_ wherein Mo exists as a monomeric species^31^), meaning that even the MoO_x_ sites in **1** are not entirely monomeric. The decrease in AEE with increasing surface density indicates a stronger interaction between the MoO_x_ species, and consequently a growth in domain size. As the coverage of the TiO_2_ support increases, Mo–O–Mo bridges form between neighbouring groups. This results in two-dimensional polymolybdates and three-dimensional MoO_3_ crystallites (Figure 9). It also narrows the HOMO–LUMO gap. Moreover, the larger domains enhance electron delocalisation, lowering the AEE. At high surface densities, the AEE values approach that of bulk MoO_3_. Hence, higher surface densities ultimately cause the conversion of part of MoO_x_ species to MoO_3_ microcrystallites. The monolayer surface density for MoO_3_ on metal oxide supports lies between 4–7 atoms/nm^2^ (based on theoretical calculations using octahedral-coordinated surface species^32^). This supports our conclusion that catalysts **3**–**5** increasingly contain three-dimensional MoO_3_ crystallites. Note that the weak band attributable to MoO_3_ was observed for higher loadings in FT-IR. Also shifts in BE of XPS spectra and *T*_max_ in TPR may indicate the increased aggregation of MoO_x_ species, forming MoO_3_ microcrystallites. However, these 3-dimensional crystallites of MoO_3_ must be below the detection limit of XRD, as no MoO_3_ peaks were detected in XRD.

### Relating Active Site Structure and Catalyst Performance

**Figure 10:**
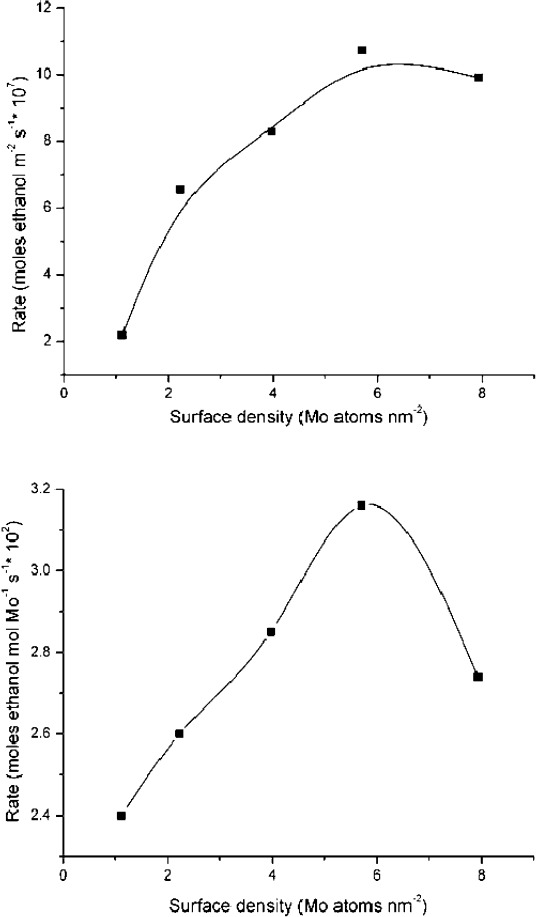
Initial rates of ethanol oxidation on MoO_x_/TiO_2_ catalysts normalised by (*top*) surface area and (*bottom*) Mo atoms, as a function of Mo atom surface density.

The fact that at 423 K the major product obtained with catalysts **1–5** was acetaldehyde, that is, a partial oxidation product, shows that redox sites predominate on these catalysts. Areal ethanol conversion rates (moles per unit area) for MoO_*x*_/TiO_2_ catalysts increase with increasing Mo-atom surface density (Figure 10, *top*), reaching a plateau at 6 Mo atoms/nm^2^. However, the rates normalised per Mo atom show a maximum at this surface density (Figure 10, *bottom*). Since the edge energy analysis showed that the surface predominantly contains two-dimensional polymolybdates below this value, this reflects the intrinsic reactivity of MoO_x_ species. As the domains grow, electron delocalisation increases. This lowers the barrier for MoO_x_ reduction. When the surface density is <6 Mo atoms/nm^2^, practically all the MoO_x_ species are accessible to the ethanol molecules. But, when the surface coverage exceeds the monolayer coverage and MoO_3_ crystallites form, some Mo atoms are inaccessible to the reactant molecules, and the rate normalised per Mo atom decreases. A similar trend was observed earlier for propane oxidative dehydrogenation.^33^ A dependence of catalytic behaviour on the surface coverage was reported for methanol oxidation on MoO_3_/TiO_2_ by Brückman et al.^34^ They found that two different zones may be distinguished which correspond to a molybdena content higher and lower than a theoretical monolayer. In a submonolayer region, the total rate of methanol consumption depends largely on the specific surface area and on the surface coverage. In the second zone, the catalytic behaviour was independent of the specific area of the support.

**Scheme 1:**
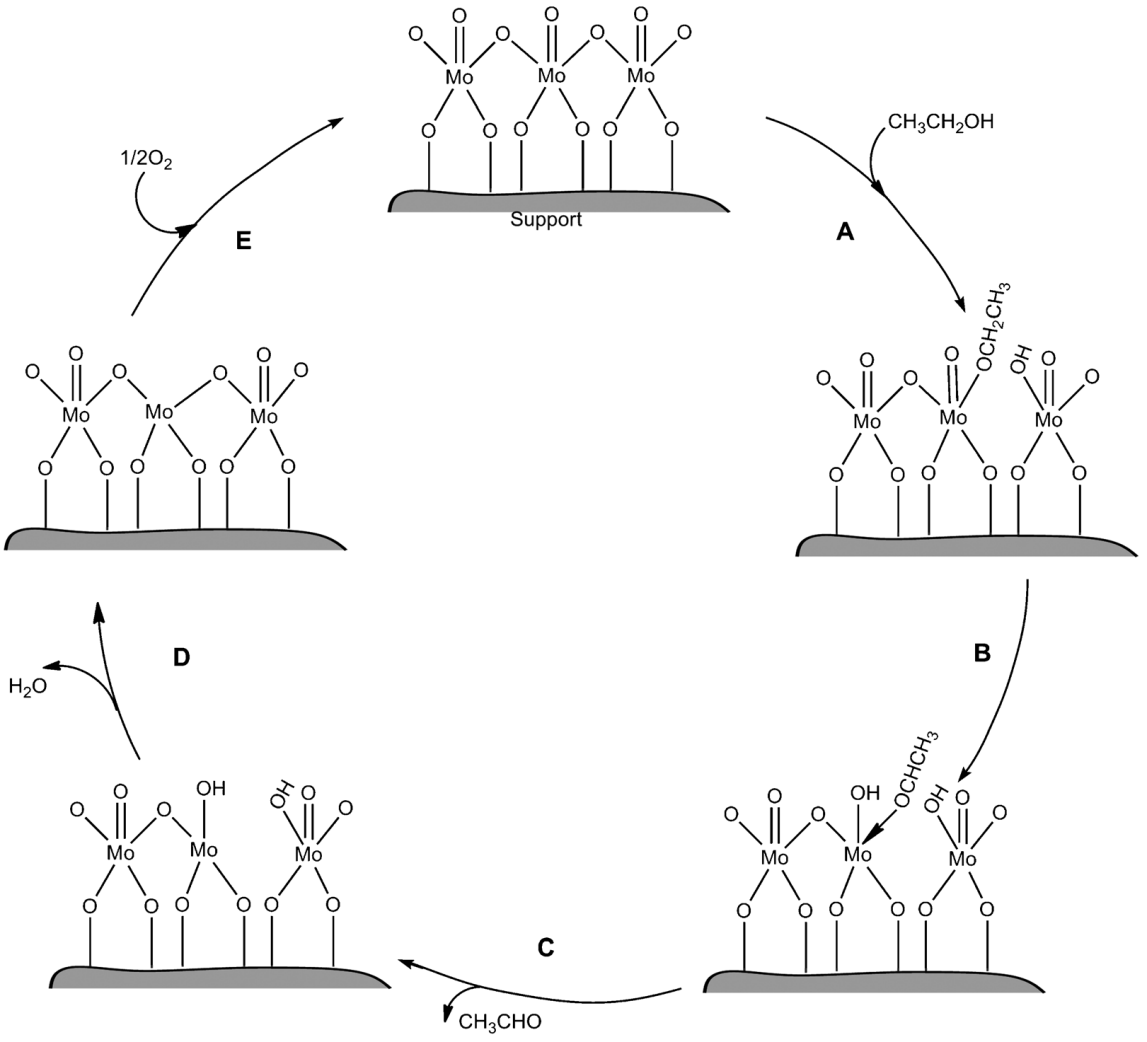
Suggested catalytic cycle for ethanol oxidation to acetaldehyde over MoO_x_-TiO_2_ catalyst.

Scheme 1 shows a possible catalytic cycle for the oxidation of ethanol to acetaldehyde.^29^,^35^ First, dissociative adsorption of ethanol on Mo centres leads to adsorbed ethoxide species and OH groups (**A**). Subsequent H-abstraction from the ethoxide species followed by desorption gives acetaldehyde (**B**, **C**). The hydroxy groups react together, giving water and Mo–O–Mo species (**D**). The oxygen vacancies, formed by dehydroxylation, are reoxidised *via* irreversible chemisorption of O_2_, closing the cycle (**E**).

Previous work on methanol oxidation suggests that reoxidation proceeds *via* O_2_ adsorption at a vacant site, forming a peroxide species.^36^ One of the two oxygen atoms fills the vacancy, while the other migrates through the near surface region until it reoxidises a second vacancy. Transient-response studies in the absence of O_2_ confirmed that ethanol oxidation to acetaldehyde indeed uses lattice oxygen atoms.^35^ The activation energy of these steps, in turn, depends on the energy required to transfer the electron from oxygen to Mo. This also determines the UV-visible absorption energy. The relationship between the rate-determining step and the photon excitation process ultimately lies here, since both are controlled by the same electronic factors. Larger domains delocalise the electrons more effectively, narrowing the HOMO–LUMO gap. This, in turn, decreases the absorption onset while increasing the reaction rate. This also explains the reciprocal relationship of the turn-over rate and AEE when all the Mo atoms are accessible to ethanol.

A report by Weber evokes a similar concept.^37^ He used MO calculations to explain the differences in rates with support by considering metal oxide-support complexes. The differences in rates were ascribed to the availability of empty electronic energy states in the metal oxide-support complex. The higher the density of empty states, the higher is the reaction rate.

## Conclusions

We have studied titania-supported molybdenum oxide catalysts for the selective oxidation of ethanol to acetaldehyde. MoO_x_/TiO_2_ catalysts are active and selective for acetaldehyde formation. The catalytic performance depends on the surface MoO_x_ species. The nature of these species is revealed by absorption edge energy values obtained by UV-Vis DRS spectral analysis and further supported by other techniques. An evolution of MoO_x_ species from monomeric to oligomeric structures with increasing surface density is apparent from the AEE values. This evolution and consequent changes in domain size determine the rate of ethanol oxidation on the various MoO_x_ domains.

## Experimental Section

### Materials and Instrumentation

Ultraviolet-visible (UV-Vis) diffuse reflectance spectra (DRS) of prepared catalysts were obtained using a UV-2450 spectrophotometer (Shimadzu) with a diffuse reflectance accessory. All samples were ground before measurements, and magnesium oxide was used as the reflectance reference. Reported spectra were taken at 300 K without any prior treatment. Previous studies suggested that *in situ* oxidation/dehydration at higher temperatures had no major effect (within the measurement limits) on absorption edge energies.^26^

The Kubelka–Munk function, F(R_∞_) for infinitely thick samples was used to convert reflectance measurements (R_sample_) into equivalent absorption spectra using MgO reflectance as a reference (R_MgO_), as shown in Eq (3).2
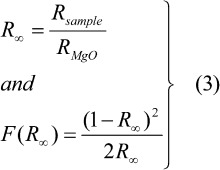


Bulk MoO_3_ was obtained from Sigma Aldrich. Ammonium molybdate tetrahydrate and titania (Eurotitania 110 m^2^ g) were obtained from Merck and Tioxide specialities Ltd. (U.K.), respectively. Ethanol (99.8%) was obtained from Royal Nedalco.

X-ray photoelectron spectroscopy (XPS) measurements were carried out using a multiprobe system (Omicron Nanotechnology, Germany) equipped with a dual Mg/Al X-ray source and a hemispherical analyser operating in constant analyser energy (CAE) mode. The spectra were obtained with pass energy of 50 eV for survey scan and 20 eV for individual scans; an Mg Kα X-ray source was operated at 300 W and 15 kV. The base pressure in the analysing chamber was maintained at 1×10^−10^ mbar. The data were processed with the Casa XPS programme (Casa Software Ltd., U.K.). The peak areas were determined by integration employing a Shirley-type background. Peaks were considered to be a mix of Gaussian and Lorentzian functions in a 70/30 ratio. For quantifying the elements, relative sensitivity factors (RSF) provided by the manufacturer were used. The peaks were calibrated by taking the adventitious carbon C 1*s* line as 284.9 eV.

X-ray diffraction (XRD) patterns were obtained using a Rigaku Miniflex II with Cu Kα radiation. The phases were identified by matching the peaks in the XRD pattern of the test samples with JCPDS (Joint Committee on Powder Diffraction Standards) data files.

Temperature programmed reduction (TPR) profiles were obtained using a Micromeritics TPD/TPR 2900 instrument. Prior to the measurements, the catalyst (∼50 mg) was dried in a TPR cell at 773 K for 2 h in a stream of He to remove water and adsorbed impurities. The TPR profiles were then obtained by passing a 10% H_2_/Ar flow (60 mL min^−1^) through the sample at temperatures from 300 to 1173 K. The temperature was increased at the rate of 10 K min^−1^ and the amount of H_2_ consumed was determined with a thermal conductivity detector (TCD). A cooling trap was placed between the sample and the TCD to retain the water produced during the reduction process.

FT-IR spectra were recorded in an IFS 66/S Bruker spectrometer with a DLATGS detector. Samples were diluted with KBr and were pressed into discs for measurements.

### Procedure for MoO_x_-TiO_2_ Catalyst Synthesis

A series of titania-supported molybdenum oxide catalysts (MoO_x_-TiO_2_) was prepared by incipient wetness impregnation of titania with aqueous solutions of ammonium heptamolybdate. Example: for 2 wt% Mo loading (catalyst **1**): 0.18 g (0.15 mmol) of ammonium molybdate tetrahydrate was dissolved in 80 mL demineralised water and stirred at 700 rpm for 5 min at 298 K. Then, 5 g of TiO_2_ were added to the solution and the suspension was stirred at 300 rpm for 5 min at 298 K. The liquid was evaporated on a rotavapor and the catalyst was dried under static air at 368 K for 12 h. The sample was then calcined at 773 K under air for 4 h. Catalysts **2**–**5**, containing 4–14 wt% of Mo, respectively, were similarly prepared.

### Procedure for Catalytic Activity Testing

**Figure 11:**
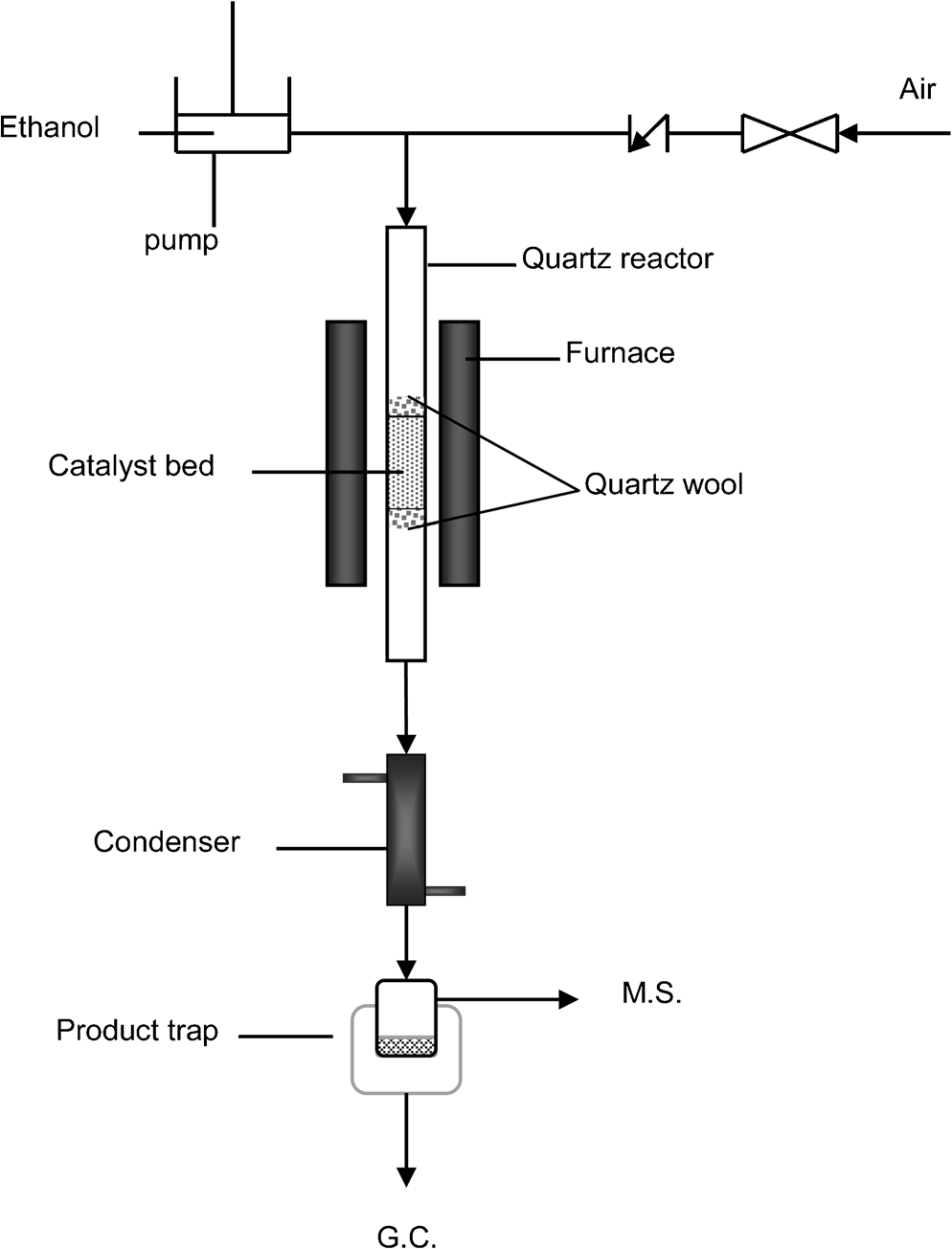
Schematic of the reactor set-up.

Reactions were conducted in a fixed-bed flow reactor (quartz with internal diameter of 4 mm) constructed in-house (Figure 11). Catalyst particles were sized between 0.5 mm and 0.9 mm. All samples were pretreated in a 50 cm^3^ min^−1^ air flow at 400 °C for 2 h to eliminate any surface moisture and to ensure complete oxidation and then allowed to cool to reaction temperature. Liquid ethanol (absolute) was vapourised into a flowing air stream by controlled injection with an ISCO 100DX syringe pump. The reactor was kept at the desired temperature using an electrical oven and a water cooling jacket. Effluents were quenched in an ice bath and products were collected and analysed by gas chromatography (GC). The analysis was performed using an Interscience GC-8000 GC with a 14% cyanopropylphenyl/86% dimethylpolysiloxane capillary column (RTX-1701, 30 m×0.25 mm, 1 μm d_f_).
